# Identification of CdnL, a Putative Transcriptional Regulator Involved in Repair and Outgrowth of Heat-Damaged *Bacillus cereus* Spores

**DOI:** 10.1371/journal.pone.0148670

**Published:** 2016-02-05

**Authors:** Alicja K. Warda, Marcel H. Tempelaars, Jos Boekhorst, Tjakko Abee, Masja N. Nierop Groot

**Affiliations:** 1 TI Food and Nutrition, Wageningen, The Netherlands; 2 Laboratory of Food Microbiology, Wageningen University, Wageningen, The Netherlands; 3 Wageningen UR Food & Biobased Research, Wageningen, The Netherlands; 4 NIZO Food Research B.V., Ede, The Netherlands; University of Connecticut, UNITED STATES

## Abstract

Spores are widely present in the environment and are common contaminants in the food chain, creating a challenge for food industry. Nowadays, heat treatments conventionally applied in food processing may become milder to comply with consumer desire for products with higher sensory and nutritional values. Consequently subpopulations of spores may emerge that are sublethally damaged rather than inactivated. Such spores may germinate, repair damage, and eventually grow out leading to uncontrolled spoilage and safety issues. To gain insight into both the behaviour of damaged *Bacillus cereus* spores, and the process of damage repair, we assessed the germination and outgrowth performance using OD_595_ measurements and microscopy combined with genome-wide transcription analysis of untreated and heat-treated spores. The first two methods showed delayed germination and outgrowth of heat-damaged *B*. *cereus* ATCC14579 spores. A subset of genes uniquely expressed in heat-treated spores was identified with putative roles in the outgrowth of damaged spores, including *cdnL* (BC4714) encoding the putative transcriptional regulator CdnL. Next, a *B*. *cereus* ATCC14579 *cdnL* (BC4714) deletion mutant was constructed and assessment of outgrowth from heat-treated spores under food relevant conditions showed increased damage compared to wild type spores. The approach used in this study allows for identification of candidate genes involved in spore damage repair. Further identification of cellular parameters and characterisation of the molecular processes contributing to spore damage repair may provide leads for better control of spore outgrowth in foods.

## Introduction

Spore forming bacteria are commonly present in the environment and difficult to eradicate because they produce highly resistant spores that may remain dormant for years until germination. The high resistance towards a diverse range of stresses make spores an important target for food industry processes aimed to produce safe, ambient stable products. Dormant spores that can be present on raw material or ingredients and survive the heat processing treatments may eventually germinate and grow out, leading to food-borne illness upon consumption of those food products or result in product spoilage. The current practice of industry is to use intense heating regimes to minimize the risk of surviving spores but consumers prefer milder processes which have less effect on sensory and nutritional values of products.

A tendency to use milder heat-treatments increases the risk of spores surviving the process and may lead to a subpopulation of spores that are sublethally damaged rather than inactivated. Sublethally damaged spores may still have the capacity to grow out if conditions allow for repair of the damage. Repair of spore damage is conceivably taking place between germination and outgrowth [1], however the processes involved in damage repair have not been studied extensively. A number of factors have been hypothesised to be involved in spore damage repair. Firstly, dormant spores may be equipped with transcripts resulting from late sporulation processes that on the one hand could support early repair of damage accumulated during dormancy, or alternatively, could serve as a reservoir of nucleotides in the germination process [1–5]. Secondly, spore damage repair may involve known repair systems for DNA damage such as AP endonucleases (Nfo and ExoA) or nucleotide excision repair enzymes (UvrA) described for *Bacillus subtilis* [1,6–9]. Spore DNA damage may accumulate during dormancy and (sub)lethal processing treatments with subsequent outgrowth requiring the activation of DNA repair systems. Despite the possible impact of spore damage repair on subsequent spore outgrowth and associated food quality and safety issues, the frequency and underlying mechanisms of this phenomenon have gained limited attention up to now [10].

The events associated with spore germination appear to occur via a tightly controlled spore outgrowth program [1,5]. Transcriptomic approaches have been performed to understand the processes and genes involved in the wake up of dormant spores and resumption of metabolic activity for the Bacillus genus [1,5] and in Clostridia [2–4]. A common finding among those studies is that mRNA levels of the majority of genes on the chromosome increase rapidly during the initial germination processes showing a highly dynamic expression pattern. Transcriptome analyses of spore germination and outgrowth performed so far, predominantly involved the use of optimal conditions in nutrient-rich media at neutral pH values. Few studies, including van Melis et al. [5] for *B*. *cereus* spores, focus on gene expression during germination and outgrowth under less favourable conditions such as presence of the preservative sorbic acid in mildly acidic conditions. Nevertheless, suboptimal conditions are typically encountered in practice in processed foods, for example when spores are damaged upon exposure to heat, and their fate is influenced by matrix composition, temperature and/or pH.

In this study, we focus on germination and outgrowth of heat-damaged spores of *B*. *cereus*, a microorganism that has been associated with food spoilage [11] and food-borne disease [12]. *B*. *cereus* associated diseases are usually mild and self-limiting but in rare instances fatal outcomes have been reported [12–17]. The vegetative cells of *B*. *cereus* can cause disease either by the production of a heat-stable toxin (cereulide) in food before ingestion resulting in emetic syndrome or by secretion of enterotoxins in the small intestine, causing the diarrheic syndrome. We assessed the germination and outgrowth performance of untreated and heat-damaged *B*. *cereus* spores using optical density measurements and microscopy analysis at selected time points. Transcriptome profiling was used to identify genes and putative molecular mechanisms involved in the repair and recovery of heat-damaged spores. To validate this approach, one candidate gene with a potential role in recovery and repair of outgrowing damaged spores was selected for mutant construction and subsequent phenotype analysis. The resulting targeted deletion mutant, *ΔcdnL* (BC4714), showed a higher fraction of severely damaged spores compared to wild type. The work presents the feasibility of the applied approach for identification of novel cellular parameters involved in repair and recovery of heat-damaged spores.

## Materials and Methods

### Strain and Sporulation Conditions

*B*. *cereus* ATCC 14579 was obtained from the American Type Culture Collection (ATCC) and routinely cultured in Bacto Brain Heart Infusion broth (BHI; standard BHI media contains 0.5% NaCl; Beckton Dickinson, Le Point de Claix, France) at 30°C with aeration at 200 rpm. Spores were prepared in a nutrient-rich, chemically defined sporulation medium (MSM medium) described previously [18]. The sporulation process and handling of resulting spores were performed as described previously [19], briefly one ml of an overnight-grown pre-culture was used to inoculate 100 ml of MSM media in 500 ml flasks and incubated at 30°C with aeration at 200 rpm. Sporulation was monitored over 2–3 days by phase contrast microscopy until over 99% of the spores were released from the mother cell. Spores were then harvested by centrifugation at 5000 rpm at 4°C (5804R, Eppendorf, Germany) for 15 min and washed with chilled phosphate buffer (100 mM, pH 7.4) containing 0.1% Tween80 to prevent spore clumping. Spores were washed twice a day for 2 weeks with a phosphate buffer that was gradually decreased in Tween80 concentration until a final concentration of 0.01% (further referred as suspension buffer). Spore suspensions free of vegetative cells and debris were stored at 4°C and used within six months.

### Heat-Treatment

A 120 μl aliquot of the spore suspension containing approximately 1X10^8^ spores/ml in suspension buffer was transferred into capillary tubes (Micropipettes 200μl max, Blaubrand intraMARK, Germany) and heat-sealed at both ends. The capillary tubes were placed either on ice or in a 95°C oil bath (Julabo MC-12, Germany) for 1 min and immediately cooled in ice-cold water. The heat-treated spore suspension was recovered from the capillary tubes and directly decimally diluted in suspension buffer. For several spore-formers, including *B*. *cereus*, *B*. *subtilis*, *Bacillus stearothermophilus*, *Clostridium* spp addition of stressful substances such as sodium chloride has been used to assess spore damage, as sublethally injured spores appear to have an increased sensitivity to those substances [20–24]. 5.5% NaCl was added to BHI, resulting in 6% final concentration, which did not affect outgrowth of untreated spores as indicated by Cazemier et al. [23] and own data ([19], S4 Table). A dose dependent sensitivity of damaged spores towards salt can be used to differentiate between severely and mildly damaged spores. Based on preliminary experiments we selected supplementation with 1.5% NaCl as an intermediate cut off providing sufficient resolution to evaluate different degrees of damage for the heat-treated spores. In short, 50 μl of serially diluted samples were plated in duplicate on BHI plates and BHI plates supplemented with 1.5% and 5.5% salt followed by incubation up to seven days at 30°C. To evaluate possible delay in colony formation, colonies were counted after 1, 2 and 7 days (further extension did not affect colony counts). Obtained colony forming units (cfu‘s) were used to calculate the total damage as reported previously [19] (see formula below). Fractions of mildly and severely damaged spores were calculated using the following formulas:

$$\%\;\mathrm{Total damage}=\frac{\left(\text{Number of cfu}’\text{s BHI})-\;(\text{Number of cfu}’\text{s BHI}\;5.5\%\;\text{NaCl}\right)}{\left(\text{Number of cfu}’\text{s BHI}\right)}\;*\;100$$

$$\%\;\mathrm{Mild damage}=\frac{\left(\text{Number of cfu}’\text{s BHI}\;1.5\%\;\text{NaCl})-\;(\text{Number of cfu}’\text{s BHI}\;5.5\%\;\text{NaCl}\right)}{\left(\text{Number of cfu}’\text{s BHI}\right)}\;*\;100$$

$$\%\;\mathrm{Severe damage}=\frac{\left(\text{Number of cfu}’\text{s BHI})\;-\;(\text{Number of cfu}’\text{s BHI}\;1.5\%\;\text{NaCl}\right)}{\left(\text{Number of cfu}’\text{s BHI}\right)}\;*\;100$$

### OD_595_ Measurement and Microscopy

To initiate spore germination and outgrowth, 180 μl of 1.1 times concentrated BHI was added to the wells of a 96 –well plate containing either 20 μl of untreated or heat-treated spore suspension containing approximately 1X10^8^ spores/ml resulting in a final concentration of 1X10^7^ spores/ml. Plates were immediately transferred to a plate reader (Tecan Infinite F200 Pro, Austria) for incubation at 30°C at approximately 200 rpm. OD_*595*_ was measured every 10 min and read outs were used to calculate the relative change in OD_595_. Duplicate plates were incubated at 30°C at approximately 200 rpm (IKA KS 250, Germany), allowing for microscopic observations and imaging at regular intervals.

### Sampling for Microarray and qPCR Experiments

Samples for RNA isolation were gathered from germinating and outgrowing spores in BHI after 10 (t10), 20 (t20), 30 (t30) and 50 (t50) min for untreated spores and 50 (t50), 90 (t90), 120 (t120) and 150 (t150) min for heat-treated spores. The time points were selected based on microscopic observations and the relative change in OD_*595*_. Eight hundred μl of concentrated spore suspension containing approximately 1X10^10^ spores/ml was injected into either pre-heated (95°C; oil bath) or ice cold glass tubes filled with 5 ml suspension buffer. After 1 min, the content of the tube was cooled by mixing with 40 ml of ice cold suspension buffer, followed by centrifugation (5804R, Eppendorf, Germany) for 5 min at 5000 rpm at 4°C and resuspension in 8 ml of suspension buffer. Two ml of this spore solution was used to inoculate four flasks with 18 ml of 1.1x BHI reaching a final concentration of 1X10^8^ spores/ml. Flasks were then incubated at 30°C with aeration at 200 rpm. At each sampling point, the content of one flask was rapidly pelleted by centrifugation (5804R, Eppendorf, Germany) at maximum speed at 4°C for 30 s and the resulting pellet was resuspended in 1 ml TRI-reagent (Applied Biosystems, United Kingdom) and rapidly frozen in liquid nitrogen. The frozen samples were stored at −80°C until RNA extraction. Sampling was performed from two independent experiments. Samples were collected for microarray analysis from the first spore batch and to support the specific expression of genes in heat-treated spores the samples for qPCR experiments were collected from a second spore batch that was prepared independently.

### RNA Isolation for Microarray and qPCR Experiments

RNA was extracted from the samples in TRI-reagent by defrosting on ice followed by mechanically disrupting the spores by exposing them to 6 rounds of 45 s of bead beating (FastPrep-24, MP Biomedicals, Germany) at maximal settings in the presence of Lysing Matrix B beads (MP Biomedicals, Germany). A direct-zol RNA MiniPrep kit (Zymo Research, United States) was used according to the manufacturer’s instructions for on column RNA purification. Residual chromosomal DNA was removed from the samples by a 30 min off column treatment using the TURBO DNA-free Kit (Ambion, United Kingdom). The resulting total RNA was subsequently cleaned with RNeasy Mini Kit (Qiagen, Germany). The RNA quantity and quality were checked by UV spectroscopy (Biophotometer, Eppendorf, Germany) and by analysis on a RNA 6000 Nano chip (Agilent, United States). Examples of RNA profiles are shown in S1 Fig. The RNA samples were stored in 70% ethanol with 83 mM sodium acetate buffer (pH5.2) at −80°C.

### cDNA Synthesis, Labelling and Microarray Hybridization and Design

Fluorescently-labelled cDNA was prepared from the extracted RNA following an indirect labelling approach with amino-allyl-labelled dUTP (Ambion, United Kingdom) and Superscript III (Invitrogen, The Netherlands) as described previously [25]. Two hundred ng of appropriately Cy3 and Cy5 -labelled cDNA was used for each sample hybridization. For each time point, independent biological duplicates were used in combination with a dye-swap approach. Array hybridisation followed a loop design (S2 Fig). Hybridization and removal of the unbound cDNA was performed as described previously [5]. The microarrays used in this study were custom-made *B*. *cereus* microarrays (8 × 15 K, Agilent, GEO accession number GPL9493, 3^rd^ design) based on *B*. *cereus* ATCC 14579 genome sequence (NCBI accession number NC_0044722).

### Microarray Scanning and Data Analysis

The microarray slides were scanned using an Agilent microarray scanner (G2565BA), and the raw data were extracted using Agilent’s Feature Extraction software (version 10.7.3.1). Microarrays were normalized using the approach reported for germinating spores involving the creation of so called synthetic microarrays [5]. Namely, the background-corrected, raw signals (Cy3 and Cy5 channel) of all arrays were hierarchically clustered (Pearson correlation, complete linkage) using Genemaths XT (version 1.6.1, Applied Maths, Belgium). New synthetic arrays (S1 Table) were defined from sample pairs showing the highest similarity in the clustering. In a next step, the synthetic microarrays were normalized (Lowess normalisation), based on normalised values three experimental samples were excluded (see S1 Table). Gene expression levels were calculated relative to that of untreated samples after 10 min (t10). This approach was chosen to exclude genes massively expressed as part of the germination programme (as observed by Melis et al. [5]) and focus on genes specifically expressed in heat-treated spores in the phase after germination and before outgrowth. Genes were included for further analysis when the following criteria were met: log2 values should be higher than 1 or lower than -1, with a false discovery rate (FDR) smaller than 0.05 in at least one time point.

To select for candidate genes uniquely expressed in heat-damaged spores (and potentially involved in heat-damage repair) the following three criteria should be met: i. upregulation (log2 above 1; FDR < 0.05) at all-time points relative to t10 for heat-treated spores, ii. downregulation (log2 below -1; FDR < 0.05) at all time points relative to t10 for untreated spores, and iii. confirmation of expression of genes selected after step i and ii, using qPCR (see section 2.8).

Special attention was given to dormant spore transcripts, putative DNA damage repair genes, and novel genes involved in damage repair. Expression of spore specific mRNAs reported for *B*. *cereus* [5] was analysed for untreated and heat-treated spores, and ratios over germinated untreated spores (t10) and heat–treated spores (t50) were plotted. Furthermore, genes known to be involved in DNA damage repair in *B*. *subtilis* were extracted from *Subti*Wiki [26] and supplemented with *B*. *cereus* ATCC 14579 genes with predicted roles in DNA repair based on their annotation (S2 Table).

### Microarray Accession Number

The data discussed in this publication have been deposited in NCBI's Gene Expression Omnibus [27] and are accessible through GEO Series accession number GSE73043 (http://www.ncbi.nlm.nih.gov/geo/query/acc.cgi?acc=GSE73043).

### qPCR

RNA was isolated from untreated (t10, t30) and heat-treated (t120) germinating and outgrowing spores in BHI as described above. Primers (S3 Table) were designed targeting the 21 selected genes that were specifically expressed in outgrowth of heat-damaged spores and five candidate genes for normalisation (BC0257, BC0544, BC1409, BC4471, and BC4743) were selected based on their constant expression levels across the different conditions used on the DNA microarrays. Due to the high number of target genes, two mixes (Mix A and Mix B) were used for first-strand cDNA synthesis using the SuperScript III reverse transcriptase (Invitrogen, United States). Two hundred ng of total RNA, 0.25 μM (final concentration) of each gene-specific reverse primer (Mix A 16 genes, Mix B 15 genes) and 0.5 mM (final concentration) of dNTP in a total volume of 14.5 μl were incubated for 5 min at 65°C and chilled on ice. After addition of 4 μl of First Strand Buffer, 1 μl of DTT (0.1 M) and 1 μl of SuperScript reverse transcriptase III (200 units/μl), the reaction was incubated for 1 h at 55°C and, finally, for 15 min at 70°C. The obtained cDNA was diluted 1:10 before use in real-time PCR.

qPCR was performed in a total volume of 25 μl using a C1000 Touch Thermal cycler (BioRad, CFX 96 Real-Time Systems). A final concentration of 0.2 μM of each primer, 12.5 μl of Power SYBR Green (Applied Biosystems, United Kingdom) and 5 μl of diluted cDNA was used as a template. The optimal annealing temperature was established at 61°C in a pre-test for primer efficiencies. The qPCR program included an initial 10 min polymerase activation step at 95°C followed by 40 cycles of denaturation (15 s at 95°C) and annealing/extension (1 min at 61°C). For each run the melting curve was checked to assure amplification of the correct target. The efficiency of the primer combinations were checked and showed good performance.

For the selection of suitable normalisation genes, two cDNA primer mixes were analysed separately. All genes in each mix were considered as potential normalisation genes and were evaluated according to the criteria defined by GeNorm [28]. Expression within Mix A was normalised with three (V3/4 = 0.142) highly stable (M<0.5) genes BC0854, BC1409 and BC4471. Expression within Mix B was normalised with five (V5/6 = 0.137) stable genes BC0257, BC0544, BC3391, BC3991, and BC4471. Normalised values were represented in relation to untreated t10 and fold change in expression was plotted. Candidate repair genes were selected based on upregulation in heat-treated spores and downregulation in untreated spores, relative to t10.

### Deletion Mutant Construction

A targeted mutant was constructed for a candidate gene involved in outgrowth of heat-damaged spores designated Δ*cdnL* (BC4714), using the temperature-sensitive plasmid pMAD [29]. To this end, a chloramphenicol resistance cassette was amplified from plasmid pNZ124 using primers CM_fwd and CM_rev (S3 Table), and the resulting fragment was digested with restriction enzymes KpnI and XhoI. The 1.3 kb flanking regions of the BC4714 gene were amplified using primers BC4714_up_fwd and BC4714_up_rev for upstream (fragment A) and, BC4714_down_fwd and BC4714_down_rev for downstream flanking regions (fragment B) (S3 Table). The resulting fragments were digested with KpnI for fragment A and with XhoI for fragment B. Fragment A, fragment B and the digested chloramphenicol resistance cassette were ligated overnight at 16°C using T4 DNA ligase. The expected 3.6 kb amplicon encompassing fragment A, B and the cassette was obtained by PCR amplification (with primers BC4714_up_fwd and BC4714_down_rev) directly on the ligation mixture. The resulting purified PCR product and pMAD plasmid were digested with EcoRI and ligated together using T4 DNA ligase. The resulting vector (plasmid pBC002), was transferred into competent *E*. *coli* TOP10 (Invitrogen) cells and four erythromycin and chloramphenicol resistant clones were selected. The presence of the correct insert was checked by PCR using primer combinations BC4714_up_fwd / BC4714_down_rev, CM_rev / BC4714_down_rev and CM_fwd / BC4714_up_fwd. Plasmid pBC002 was purified using the Maxiprep Kit (Qiagen) and was transferred into electroporated (400Ω, 25 μF, 1.2 kV) *B*. *cereus* ATCC 14579 cells as described previously [30], followed by plating on Luria Bertani (LB) agar with 5 μg/ml chloramphenicol (Cm5) and 3 μg/ml erythromycin (Ery3). A single colony harbouring plasmid pBC002 was inoculated in LB broth without antibiotics and incubated at 39°C overnight. The culture was diluted 100 fold in fresh LB medium without antibiotics and propagated for 17 generations at 39°C. Appropriate dilutions were plated on LB with Cm5 and incubated overnight at 37°C to obtain single colonies. Resulting colonies were replica plated on LB with Cm5 or Ery3 and incubated at 37°C overnight. Candidates were selected based on chloramphenicol resistance and erythromycin sensitivity resulting from desired double cross over events. Five candidate colonies displaying the desired Cm-resistant and erythromycin-sensitive phenotype were verified by PCR using primer combinations BC4714_mu_up / Cm_fwd and BC4714_mu_down combined with Cm_rev and resulted in expected fragment lengths of 2.7 and 2.6 kb, respectively. Correct disruption of the gene was further confirmed by DNA sequencing.

### Phenotype of a *cdnL* Mutant

Spores of the deletion mutant were prepared and heat-treated as described in paragraphs 2.1 and 2.2. Additionally, wild type and deletion mutant spores were exposed for 5 min to oxidative stress using hydrogen peroxide (5%; Merck) or sodium hypochlorite (0.002% active chlorite; Sigma Aldrich). One hundred μl of concentrated disinfectant solution was added to 400 μl of spore suspension containing approximately 1.2X10^8^ spores/ml. After 5 min incubation, 100 μl was transferred to 900 μl of inactivation solution containing respectively catalase solution (500 U/ml; Sigma) or sodium thiosulphate (10 g/L; Merck). This 10 min inactivation step was followed by serial dilution in suspension buffer and plating. Survival and degree of damage of heat, hydrogen peroxide and sodium hypochlorite treated spores were evaluated as described above. Experiments were performed in triplicate at room temperature.

## Results and Discussion

### Impact of Heat-Treatment on Germination and Outgrowth

The impact of heat-treatment on germination and outgrowth of *B*. *cereus* ATCC 14579 spores was assessed using spores in suspension buffer heated for one min at 95°C. This heat-treatment resulted in 2 log inactivation with a significant fraction (>90%) of damaged spores among the survivors (data not shown) which agrees with our previously reported data [19].

**Fig 1:**
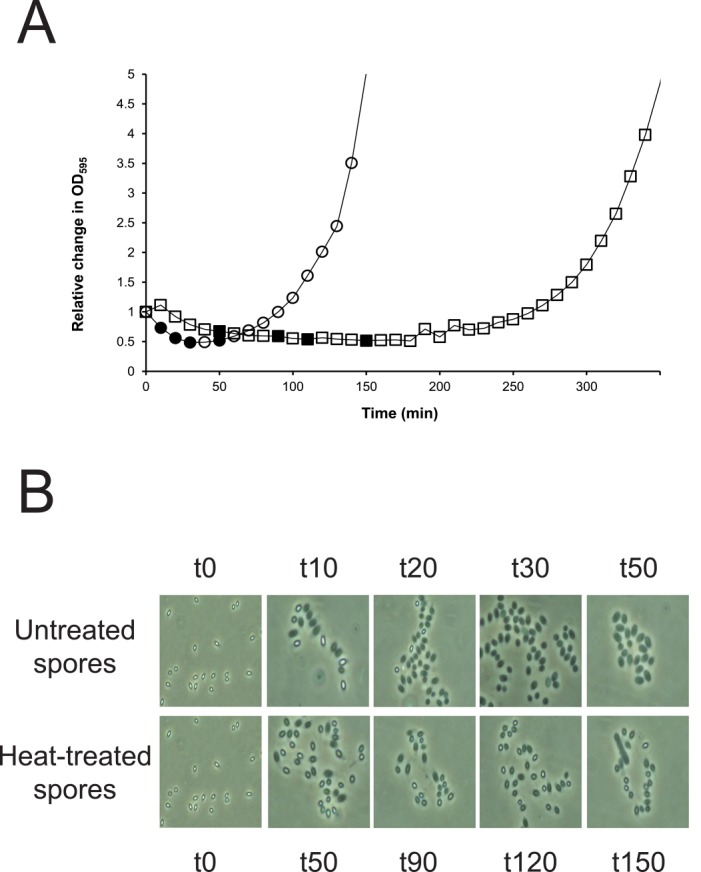
Impact of heat-treatment on germination and outgrowth of *B*. *cereus* ATCC14579 spores. (A) Relative change in OD_595_ for untreated (circles) and heat-treated for 1 min at 95°C (squares) dormant spores was monitored in time in BHI broth at 30°C. Closed symbols indicate the sampling points selected for transcriptome analysis. The starting OD_595_ was 0.15–0.2 (B) Microscopy analysis of samples taken before initiation of germination (t0) and at indicated time points (10 up to 150 min) thereafter.

Germination and outgrowth of untreated and heat-treated spores was monitored by microscopic observation and by the relative change in optical density at 595 nm (OD_595_) that reflects the transition from phase bright to phase dark spores. For the untreated spores, a rapid drop in OD_595_ was observed within 30 min from the addition of BHI (Fig 1A) resulting from the uptake of water and this coincided with the presence of phase dark spores observed by microscopy (Fig 1B). After the initial rapid drop, indicating homogeneous germination (Fig 1B), the OD_595_ increased corresponding with spore outgrowth.

Compared to untreated spores, heat-treated spores showed a delayed drop in OD_595_ as well as slower decrease in OD_595_ corresponding to a delayed germination process (Fig 1A). These observations were in line with microscopy observations (Fig 1B), and were conceivably caused by the presence of a high number of heat inactivated spores that became permeabilised and susceptible to water influx as shown previously [19]. The majority of heat-treated spores showed a slow peripheral loss of spore brightness, eventually reaching phase grey within the time frame of the experiment (Fig 1B). The number of spores showing this behaviour corresponded to the presence of 2 logs of inactivated spores as determined by plate counting. The remaining population completed germination and outgrowth though the whole process was delayed, slower and more heterogeneous compared to untreated spores (Fig 1B). Eventually, rapid exponential growth of surviving germinated outgrown spores for both untreated and heat-treated spores was observed (Fig 1A).

A number of studies have described the effect of heat-treatment on germination efficiency and/or outgrowth capacity of dormant spores of Bacilli [31–33] and Clostridia [34]. As expected, the intensity of the heat-treatment has a large effect on the behaviour of treated spores, i.e., milder treatments aiming at spore activation resulted in faster germination and outgrowth, while more intense treatments resulted in inactivation and extended times to outgrowth of surviving spores suggesting spore damage.

The heat-damaged spores conceivably activate repair mechanisms leading to recovery and subsequent vegetative growth at growth rates comparable to unstressed populations [34,35]. It is generally assumed that damage repair takes place between germination (and resumption of metabolic activity) and outgrowth during transition phase (by some authors referred to as ripening time) [34,36]. Recent findings point to synthesis of a subset of proteins during early germination [37] but the majority of the proteins were synthesized during the transition phase and early outgrowth and therefore we chose to focus on this transition stage. An additional argument is that spores with partly degraded and/or damaged mRNAs displayed an extended transition phase leading to delayed outgrowth [38,39] which also suggests that repair processes may take place during this stage.

### Transcriptome Analysis of Outgrowth from Heat-Treated Spores

A transcriptome analysis was performed for untreated and heat-treated spores aiming at identification of genes uniquely expressed during the transition phase and early outgrowth in heat-treated spores. Because the timeline of spore germination and outgrowth phases varied between untreated and heat-treated spores, we selected time points for microarray sampling based on microscopy and OD_595_ measurements thereby aiming for conditions representing comparable stages in the recovery phase. Based on data presented in Fig 1, untreated spores were sampled at time points 10, 20, 30 and 50 min after addition of BHI, and heat-treated spores were sampled at time points 50, 90, 120 and 150 min. The time point at 10 min after addition of BHI (t10) was selected as a reference point since a difference in responses was expected in transition phase and not in the germination phase where the majority of the genes are expressed (as observed by Melis et al. [5]). Even with these procedures a high number of genes was (temporarily) affected, with at least 1000 genes being differently expressed at each time point relative to t10 (data not shown). For heat treated spores, this number was twice as high (data not shown); this increase likely reflects a delay in the germination and outgrowth process but may also include genes specifically expressed to repair damage. Various studies show that spore germination and outgrowth are complex and tightly regulated processes [1,36,37]. Dormant spores contain a limited number of transcripts, typically 46 transcripts are present in the dormant spores of *B*. *cereus* [5] but upon germination 80% of the genomic content of the dormant spore is expressed. Previous studies in *B*. *subtilis* [1] and *Clostridium difficile* [4] presented gene expression of germinating spores relative to that of mid exponential cells to identify genes specifically expressed in outgrowing spores showing that 27% and 14% of the genes were uniquely expressed at one or more points during outgrowth, respectively. A common finding among all those approaches is that expression of a large proportion of the genome is initiated during germination and outgrowth, resulting in expression of genes required to resume metabolic activity including transcription, translation and metabolic activities [1,4,5].

#### Spore specific transcripts

**Fig 2:**
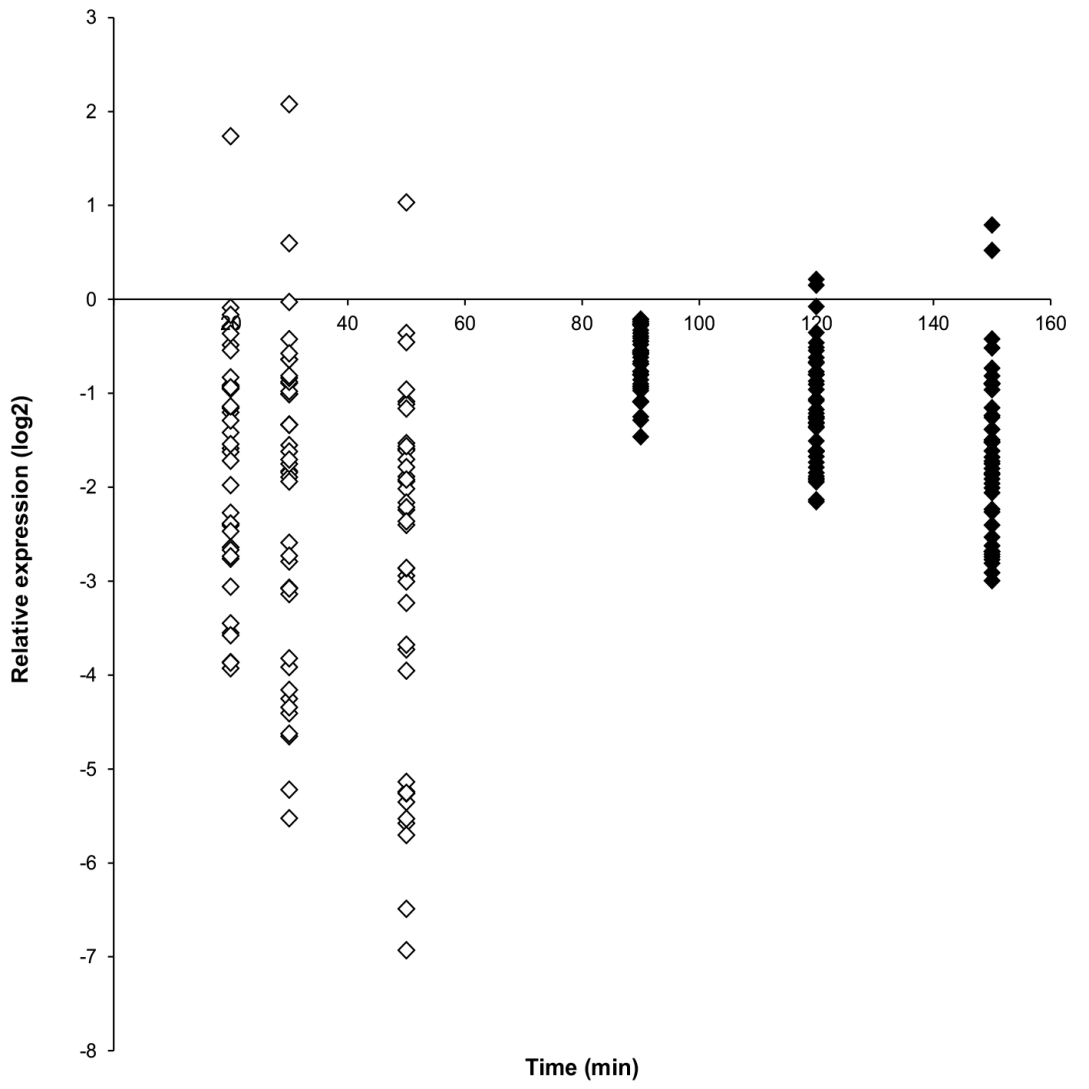
Expression profiles (log2 values) of reported spore specific transcripts during germination and outgrowth of untreated (white diamonds) and heat-treated (black diamonds) *B*. *cereus* ATCC14579 spores. Expression ratio’s for untreated spores are relative to untreated germinating control spores at t10, and for heat-treated spores relative to heat-treated germinating spores at t50.

Spores contain a relatively small set of transcripts, and for *B*. *cereus* [5] and *B*. *subtilis* [1], 46 and 23 transcripts have been identified, respectively. These transcripts, that encode hypothetical proteins and some proteins associated with spore coat composition, are rapidly broken down upon germination [1,5] suggesting that this phenomenon can be used as a marker for onset of germination. In the current study, 41 of the 46 spore transcripts reported previously for *B*. *cereus* by van Melis et al. [5] were identified. However, the level of spore transcripts generally decreased rapidly upon germination of untreated spores, while spore transcripts in the heat-treated spores decreased at a slower rate (Fig 2) being in line with the presumed delayed turnover of the mRNAs in spores arrested in germination [5]. Based on these data, the spore transcripts were not selected for further study.

#### Expression analysis of putative DNA damage repair genes

Transcriptomes of untreated and heat-treated spores were analysed for expression of known DNA repair genes reported in the literature for *B*. *subtilis* and/or present in *B*. *cereus* (S2 Table) [1,9,40]. During the sporulation process, spores may be equipped with DNA repair enzymes that allow for fast repair upon germination [8]. In addition, genes encoding DNA repair enzymes may be activated during germination and outgrowth of heat-damaged spores.

During germination and outgrowth of untreated and heat-treated spores, 49 putative DNA repair genes were differently expressed at least at one time point including 33 genes that also showed expression during outgrowth of untreated spores. Previous studies in *B*. *subtilis* showed significant number of these genes to be differently expressed during spore germination and outgrowth including *addAB*, *urvA*, *mutS*, *nth* and *nfo* [1]. Notably, both untreated and heat-treated *B*. *cereus* 14579 spores display a similar expression pattern for the DNA repair genes during germination and outgrowth albeit that the response was delayed in the latter case. A recent study in *B*. *subtilis* shows that DNA repair and outgrowth processes may be aligned to each other mediated by a specific DNA integrity scanning protein (DisA), that was found to delay spore outgrowth until oxidative DNA damage is repaired [7]. Both untreated and heat-damaged *B*. *cereus* spores expressed *disA* during germination (data not shown). Since expression patterns of DNA repair genes did not meet the criteria set in the current study they were not selected for further analysis. Nevertheless, lack of differential expression of known DNA repair genes in outgrowing heat-treated spores is in line with previously reported data [10,41–43] suggesting that wet heat treatment leads mainly to protein damage, in contrast to dry heat treatment that causes DNA damage. However, the exact effect of protein damage and repair processes involved remain to be elucidated.

#### Novel genes involved in spore damage repair

Transcriptome data were screened for genes specifically upregulated in heat-treated spores following the criteria defined in the Materials and Methods section. Expression of 21 genes that met the initial criteria was verified by qPCR using independently prepared spore batches (S3 Fig) to confirm genes specifically expressed in heat-treated spores. Using this criterion, 8 genes were selected that displayed the desired expression profile, i.e., gene expressed in heat-treated spores while downregulated in untreated spores (see Table 1).

**Table 1:** Array expression ratios (log2 values) of candidate genes displaying specific upregulation during germination and outgrowth of heat-treated *B*. *cereus* ATCC14579 spores and verified by qPCR. False Discovery Rates below 0.05 are indicated in bold.

Gene	Function	Log2 values over T10 untreated						
		Untreated			Heat-treated			
		T20	T30	T50	T50	T90	T120	T150
BC1312	3-hydroxybutyryl-CoA dehydratase	**-1.39**	**-1.34**	**-1.29**	**3.49**	**3.73**	**3.49**	**2.93**
BC3437	cytoplasmic protein	**-1.18**	**-1.34**	**-1.97**	**3.91**	**4.65**	**4.36**	**3.63**
BC3438	PadR family transcriptional regulator	**-1.09**	**-1.25**	**-1.97**	**4.33**	**4.96**	**4.61**	**3.72**
BC3921	hypothetical protein	**-2.31**	**-3.23**	**-3.86**	**1.39**	**1.79**	**1.64**	**1.07**
BC4714	CarD_CdnL_TRCF family transcriptional regulator	**-1.35**	**-1.66**	**-2.59**	**2.60**	**3.20**	**2.76**	**1.91**
BC4834	ArsR family transcriptional regulator	**-1.09**	**-1.29**	**-1.35**	**2.25**	**2.51**	**1.97**	**1.12**
BC5038	MarR family transcriptional regulator	**-1.82**	**-2.21**	**-2.74**	**1.50**	**2.08**	**1.68**	**1.06**
BC5242	membrane protein with C2C2 zinc finger	**-1.09**	**-1.01**	**-1.28**	**1.73**	**1.34**	**1.04**	**1.20**

The putative functions of candidate genes include transcriptional regulator (4 genes), membrane protein (2 genes), lyase and a hypothetical protein (Table 1). Interestingly, only three genes have an orthologue in *B*. *subtilis* 168 including hypothetical protein BC3921 with an N-acyltransferase motif such as in *ylbP* (BSU15100) and an ArsR family transcriptional regulator (BC4834) orthologous to SdpR, a transcriptional repressor of the *sdpR-sdpI* operon (BSU33790). The SdpR autorepressor belongs to the ArsR/SmtB family of repressors, whose prototypical member, ArsR, inhibits the transcription of genes involved in resistance to arsenate [44]. Finally, a CarD_CdnL_TRCF family transcriptional regulator (BC4714), with unknown function in *B*. *cereus* was identified. An orthologue is present in the *B*. *subtilis* genome, i.e., YdeB (BSU05130), but its role is also unknown. This candidate gene, further referred to as *cdnL* (BC4714), was also upregulated in vegetative cells of *B*. *cereus* in response to different stresses including cold shock, salt, acid and disinfectants [45]. Therefore, *cdnL* (BC4714) was selected to validate its potential role in spore damage repair. The *ΔcdnL* (BC4714) deletion mutant was constructed and spores were prepared. Wild type and *cdnL* (BC4714) mutant strain spores were heat-treated for one min at 95°C resulting in a 2 log inactivation and more than 90% of damaged spores among the surviving fraction for both wild type and *ΔcdnL* (BC4714) spores (Table 2). The heat resistance of *ΔcdnL* (BC4714) spores, and the kinetics of spore recovery (S4 Table) were comparable to that of wild type spores. However, plate counting with and without salt supplementation showed significant differences in the fraction of mildly and severely damaged spores, with wild type spores and *ΔcdnL* (BC4714) spores showing approximately 45% and 74% of severely damaged spores, respectively. The increased fraction of severely damaged spores in the mutant point to a role for *cdnL* (BC4714) in recovery of heat-damaged *B*. *cereus* 14579 spores. Deletion of *cdnL* (BC4714) did not result in increased salt sensitivity of untreated spores (S4 Table), and nor was the growth rate of vegetative cells affected in the presence of salt (data not shown) confirming the role of *cdnL* (BC4714) in recovery of heat-damaged *B*. *cereus* 14579 spores.

**Table 2:** Surviving and mildly and severely heat damaged spore fractions in *B*. *cereus* ATCC1457 and its mutant derivative strain Δ*cdnL* (BC4714) upon exposure to wet heat, hydrogen peroxide and sodium hypochlorite treatments. Averages of three independent experiments are represented.

				Damage in surviving fraction					
		Survival		Total damage		Mildly damaged spores		Severely damaged spores	
Treatment	Strain	%	SD	%	SD	%	SD	%	SD
95°C	WT	8	1	91	2	46	1	45	2
95°C	ΔcdnL (BC4714)	5	0	99	2	25	9	74	9
Hydrogen peroxide	WT	9	2	99	0	17	2	82	3
Hydrogen peroxide	ΔcdnL (BC4714)	7	3	99	1	16	11	83	11
Sodium hypochlorite	WT	7	1	98	0	25	6	74	7
Sodium hypochlorite	ΔcdnL (BC4714)	11	4	99	0	23	6	76	7

It is possible that the *cdnL* gene (BC4714) is induced in response to heat damaged proteins however in vegetative *B*. *cereus* cells, *cdnL* (BC4714) is induced upon exposure to salt and cold stress, and to a lesser extent to acid and oxidative stress, but not in response to heat [45]. The precise function of *cdnL* (BC4714) in those conditions remains to be elucidated. Heat-treatment was shown previously to cause secondary oxidative stress in *B*. *cereus* vegetative cells [46]. To elaborate on the role of *cdnL* (BC4714) in the recovery from stresses other than heat, spores were exposed to a oxidative treatment with hydrogen peroxide (HP) and a combination of oxidative and chloraminating action of sodium hypochlorite (SH). In both cases, deletion of *cdnL* (BC4714) did not result in increased inactivation or altered degrees of spore damage (Table 2). This suggests that *cdnL* (BC4714) plays a specific role in repair of heat-induced *B*. *cereus* spore damage. It cannot be excluded that *cdnL*-deficient spores lack one or more specific proteins that makes them more susceptible to heat damage, however, high upregulation of the *cdnL* gene in heat treated spores supports our finding that its activity is also required during spore outgrowth.

CdnL (BC4714) belongs to CarD_CdnL_TRCF family of bacterial RNA polymerase-binding proteins. A family archetype, CarD, is associated with carotenogenesis and fruiting body formation in *Myxococcus xanthus*, and is one of two prokaryotic examples of high-mobility group A (HMGA) proteins [47–49]. The C-terminus of CarD contains a HMGA domain responsible for DNA binding and the N-terminus contains a transcription repair coupling factor (TRCF) domain that shares a binding site with the RNAP β subunit [50]. A more common member of the bacterial CarD family is represented by the CarD N-terminal like protein (CdnL) that lacks a DNA-binding domain and is also present in *M*. *xanthus* and *Mycobacterium tuberculosis* where it has an important role in stress resistance [49]. In particular, the *M*. *tuberculosis cdnL* gene showes high up-regulation after DNA damaging and starvation treatments, indicating its possible link with damage repair [47,48,51,52]. In *B*. *cereus* strain ATCC14579, two paralogs of the *cdnL* gene (BC3648 and BC4714) are present, but both genes share limited similarity. Given differences in regulatory processes between *B*. *cereus* and *B*. *subtilis* [25,45] it is not surprising that YdeB, a *B*. *subtilis* orthologue of CdnL (BC3648 and BC4714) also shows limited similarity and is believed not be functionally equivalent. Future studies in *B*. *cereus* will assess the role of the other selected genes in spore damage repair including single and double mutant analysis of the BC3648 (paralogue) and BC4714 genes.

The high number of protection systems and spore structures involved in spore resistance stresses the importance of damage prevention for spore survival. Despite these defence systems, the occurrence of spore damage cannot be prevented and a repertoire of repair mechanisms is required, including genes involved in DNA repair [1,9,40]. The common consensus is that damage repair take place during transition phase between germination and outgrowth, however, the processes involved are still largely unknown. The existence of multiple systems acting in parallel is conceivable as we identified here 8 candidate genes with four of these having a possible regulatory role. The relatively mild effect of *cdnL* deletion could be explained by the presence of additional regulators and systems involved in repair.

In conclusion, using transcriptome analysis 8 candidate genes with putative roles in the outgrowth from heat-damaged *B*. *cereus* spores were identified. Comparative analysis of the wild type and a *cdnL* (BC4714) mutant shows that this gene is contributing to heat-induced spore damage repair whereas wild type and mutant spores displayed similar sensitivity to damage caused by oxidative agents indicating that we identified a novel player in heat-induced *B*. *cereus* spore damage repair. This work provided a new strategy to study and identify putative cellular parameters involved in spore damage repair, applicable not only for heat-induced damage but also for other food relevant conditions. Insights obtained may contribute to development of more efficient strategies to control outgrowth of damaged spores.

## Supporting Information

S1 Fig Examples of Bioanalyser RNA profiles from RNA samples collected and isolated during germination and outgrowth of untreated and heat-treated *B*. *cereus* ATCC1457 spores. (PDF)

S2 Fig Loop design used for hybridization of 200 ng of Cy3 (beginning of an arrow) and Cy5 (end of an arrow) -labelled cDNA. (PDF)

S3 Fig Real-time PCR quantification of candidate genes transcript levels from two experiments. Relative expression levels in samples of heat-treated (black) and untreated (white) *B*. *cereus* ATCC14579 spores during the germination and outgrowth, 120 and 30 minutes after addition of BHI, respectively. Expression ratios presented are relative to untreated germinating control spores at t10.
(PDF)

S1 Table Synthetic arrays were constructed based on hierarchical clustering of background-corrected, raw signals (Cy3 and Cy5 channel) of the arrays (*after normalisation samples that did not cluster correctly were excluded from further analysis). (PDF)

S2 Table Expression ratios (log2 values) of DNA repair genes during germination and outgrowth of untreated and heat-treated *B*. *cereus* ATCC14579 spores. Ratios expressed are relative to untreated germinating control spores at t10. False Discovery Rates below 0.05 are indicated in bold.
(PDF)

S3 Table qPCR and PCR primers used in this study. qPCR candidate normalization genes were selected based on stable and significant expression at all time points of the microarray. Recognition sites introduced for restriction enzymes used for mutant construction are underlined in PCR primers.
(PDF)

S4 Table Number of cfu’s/ml measured at day one, two and seven for untreated spores of *B*. *cereus* ATCC1457 and its mutant derivative strain Δ*cdnL* (BC4714) and upon exposure to wet heat, hydrogen peroxide and sodium hypochlorite treatments. Counts of three independent experiments are represented individually.
(PDF)
