# Supplementary material for: Identification of CdnL, a Putative Transcriptional Regulator Involved in Repair and Outgrowth of Heat-Damaged Bacillus cereus Spores
Source: PLoS One. 2016 Feb 5;11(2):e0148670. doi: 10.1371/journal.pone.0148670 (PMC4746229; doi:10.1371/journal.pone.0148670)
Supplement: S1 Fig — (PDF) [file pone.0148670.s001.pdf]

S1 Fig. Examples of Bioanalyser RNA profiles from RNA samples collected and isolated during germination and outgrowth of untreated and heat-treated B. cereus ATCC1457 spores

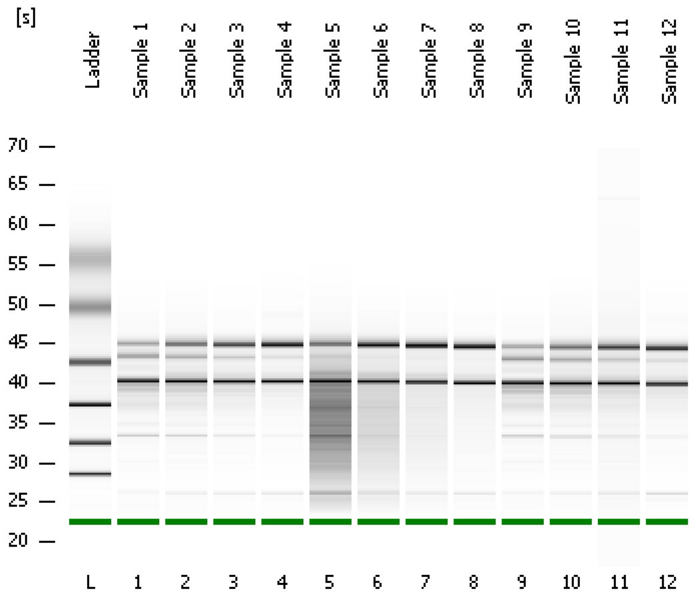

sample 1 & 9 - untreatred spores, t10
sample 2 & 10 - untreatred spores, t20
sample 3 & 11 - untreatred spores, t30
sample 4 & 12 - untreatred spores, t50
sample 5 - heat treatred spores, t50
sample 6 - heat treatred spores, t90
sample 7 - heat treatred spores, t120
sample 8 - heat treatred spores, t150
