# Supplementary material for: Identification of CdnL, a Putative Transcriptional Regulator Involved in Repair and Outgrowth of Heat-Damaged Bacillus cereus Spores
Source: PLoS One. 2016 Feb 5;11(2):e0148670. doi: 10.1371/journal.pone.0148670 (PMC4746229; doi:10.1371/journal.pone.0148670)

S2 Fig. Loop design used for hybridization of 200 ng of Cy3 (beginning of an arrow) and Cy5 (end of an arrow) -labelled cDNA

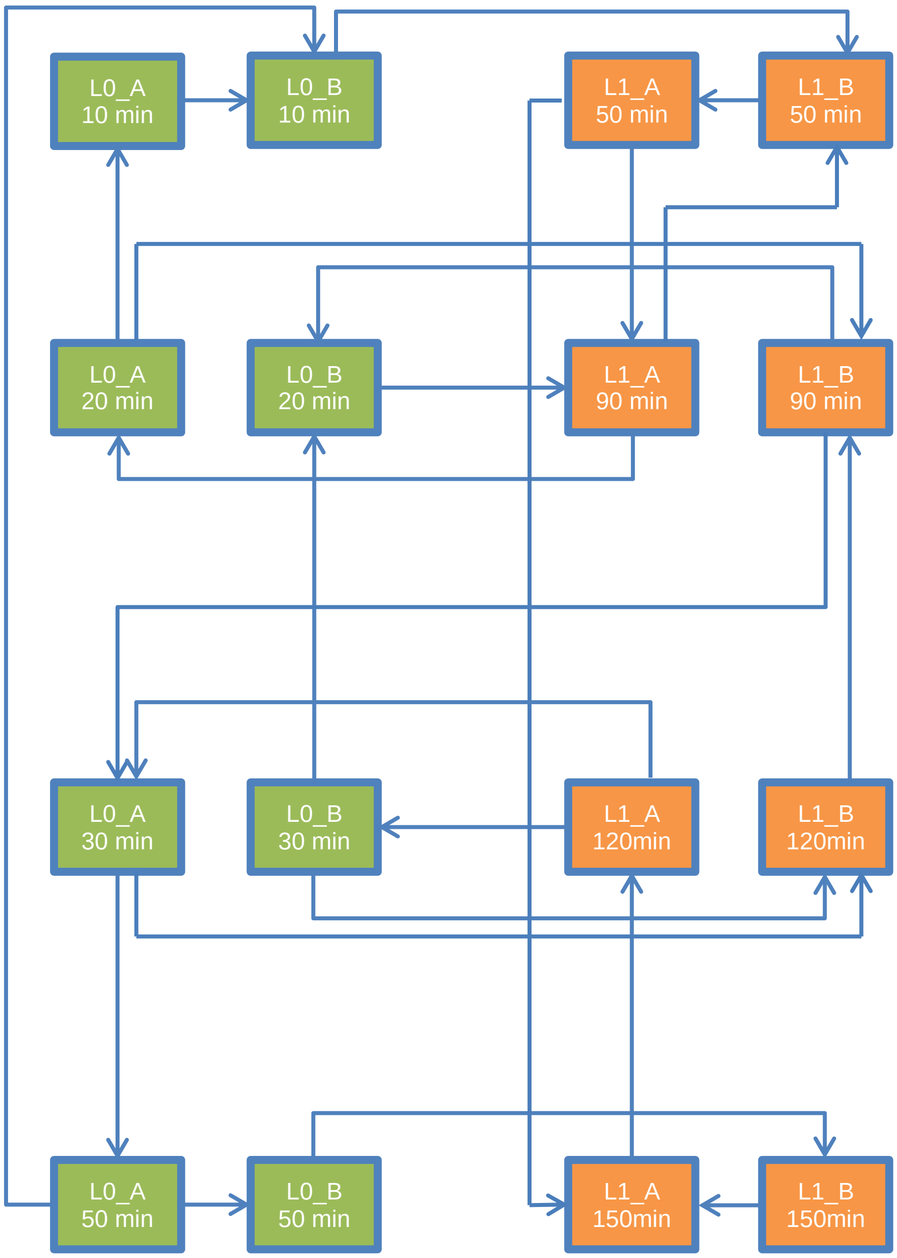

Supplement: S2 Fig — (PDF) [file pone.0148670.s002.pdf]
