# Supplementary material for: Identification of CdnL, a Putative Transcriptional Regulator Involved in Repair and Outgrowth of Heat-Damaged Bacillus cereus Spores
Source: PLoS One. 2016 Feb 5;11(2):e0148670. doi: 10.1371/journal.pone.0148670 (PMC4746229; doi:10.1371/journal.pone.0148670)

S3 Fig. Real-time PCR quantification of candidate genes transcript levels from two experiments. Relative expression levels in samples of heat-treated (black) and untreated (white) *B. cereus* ATCC14579 spores during the germination and outgrowth, 120 and 30 minutes after addition of BHI, respectively. Expression ratios presented are relative to untreated germinating control spores at t10

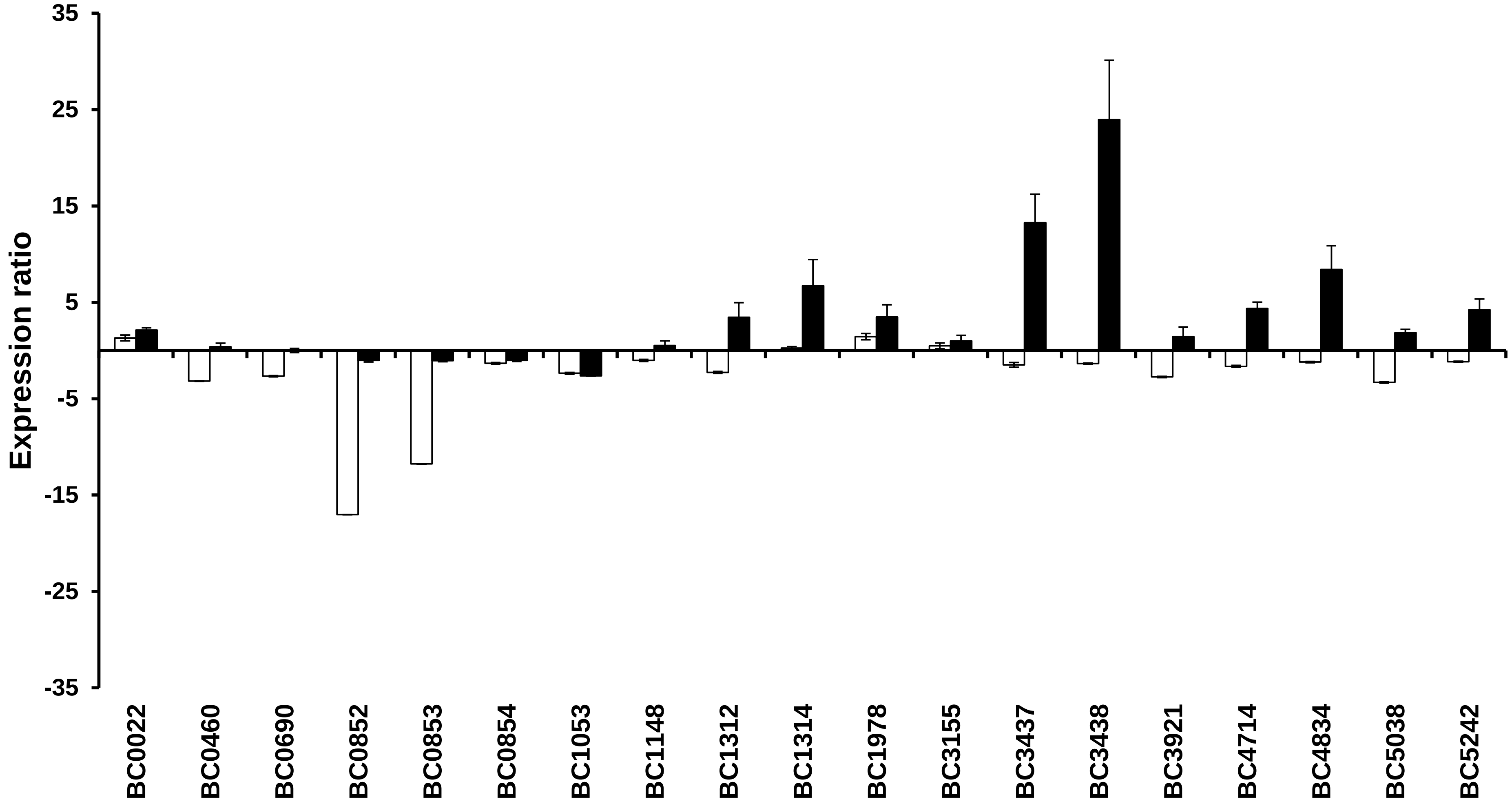

Supplement: S3 Fig — Relative expression levels in samples of heat-treated (black) and untreated (white) B. cereus ATCC14579 spores during the germination and outgrowth, 120 and 30 minutes after addition of BHI, respectively. Expression ratios presented are relative to untreated germinating control spores at t10. (PDF) [file pone.0148670.s003.pdf]
