# Supplementary material for: Identification of CdnL, a Putative Transcriptional Regulator Involved in Repair and Outgrowth of Heat-Damaged Bacillus cereus Spores
Source: PLoS One. 2016 Feb 5;11(2):e0148670. doi: 10.1371/journal.pone.0148670 (PMC4746229; doi:10.1371/journal.pone.0148670)
Supplement: S1 Table — (PDF) [file pone.0148670.s004.pdf]

S1 Table. Synthetic arrays were constructed based on hierarchical clustering of background-corrected, raw signals (Cy3 and Cy5 channel) of the arrays (*after normalisation samples that did not cluster correctly were excluded from further analysis)

RED		GREEN	
US22502548_251734310063_S02_GE2_107_Sep09_1_4%7C1.txt_Red	L0 50B	US22502548_251734310064_S01_GE2_107_Sep09_2_2%7C1.txt_Green	L0 50B
US22502548_251734310063_S02_GE2_107_Sep09_1_1%7C1.txt_Red	L0 10B	US22502548_251734310063_S02_GE2_107_Sep09_1_1%7C1.txt_Green	L0 10A
US22502548_251734310063_S02_GE2_107_Sep09_1_2%7C1.txt_Red	L0 10A	US22502548_251734310064_S01_GE2_107_Sep09_1_2%7C1.txt_Green	L0 10B
US22502548_251734310063_S02_GE2_107_Sep09_1_3%7C1.txt_Red	L0 50A	US22502548_251734310065_S01_GE2_107_Sep09_2_3%7C1.txt_Green	L0 50A
US22502548_251734310063_S02_GE2_107_Sep09_2_4%7C1.txt_Red	L0 30B*	US22502548_251734310063_S02_GE2_107_Sep09_2_4%7C1.txt_Green	L1 120A*
US22502548_251734310063_S02_GE2_107_Sep09_2_1%7C1.txt_Red	L1 90A	US22502548_251734310063_S02_GE2_107_Sep09_2_2%7C1.txt_Green	L1 90A
US22502548_251734310063_S02_GE2_107_Sep09_2_2%7C1.txt_Red	L0 20A	US22502548_251734310063_S02_GE2_107_Sep09_1_3%7C1.txt_Green	L0 30A
US22502548_251734310063_S02_GE2_107_Sep09_2_3%7C1.txt_Red	L0 30A	US22502548_251734310064_S01_GE2_107_Sep09_1_4%7C1.txt_Green	L0 30B
US22502548_251734310064_S01_GE2_107_Sep09_1_4%7C1.txt_Red	L0 20B	US22502548_251734310064_S01_GE2_107_Sep09_1_3%7C1.txt_Green	L0 20B
US22502548_251734310064_S01_GE2_107_Sep09_1_1%7C1.txt_Red	L1 120A	US22502548_251734310065_S01_GE2_107_Sep09_1_1%7C1.txt_Green	L1 120B
US22502548_251734310064_S01_GE2_107_Sep09_1_2%7C1.txt_Red	L1 50B	US22502548_251734310065_S01_GE2_107_Sep09_2_4%7C1.txt_Green	L1 50A
US22502548_251734310064_S01_GE2_107_Sep09_1_3%7C1.txt_Red	L1 90A	US22502548_251734310064_S01_GE2_107_Sep09_1_1%7C1.txt_Green	L1 150A
US22502548_251734310064_S01_GE2_107_Sep09_2_4%7C1.txt_Red	L0 20B	US22502548_251734310063_S02_GE2_107_Sep09_1_2%7C1.txt_Green	L0 20A
US22502548_251734310064_S01_GE2_107_Sep09_2_1%7C1.txt_Red	L1 120B	US22502548_251734310063_S02_GE2_107_Sep09_2_3%7C1.txt_Green	L1 120A
US22502548_251734310064_S01_GE2_107_Sep09_2_2%7C1.txt_Red	L1 150B	US22502548_251734310063_S02_GE2_107_Sep09_1_4%7C1.txt_Green	L0 50A*
US22502548_251734310064_S01_GE2_107_Sep09_2_3%7C1.txt_Red	L1 50A	US22502548_251734310064_S01_GE2_107_Sep09_2_3%7C1.txt_Green	L1 50B
US22502548_251734310065_S01_GE2_107_Sep09_1_4%7C1.txt_Red	L0 30A	US22502548_251734310065_S01_GE2_107_Sep09_2_1%7C1.txt_Green	L0 30A
US22502548_251734310065_S01_GE2_107_Sep09_1_1%7C1.txt_Red	L1 90B	US22502548_251734310064_S01_GE2_107_Sep09_2_4%7C1.txt_Green	L1 90B
US22502548_251734310065_S01_GE2_107_Sep09_1_2%7C1.txt_Red	L1 150A	US22502548_251734310065_S01_GE2_107_Sep09_1_2%7C1.txt_Green	L1 150B
US22502548_251734310065_S01_GE2_107_Sep09_1_3%7C1.txt_Red	L1 90B	US22502548_251734310063_S02_GE2_107_Sep09_2_1%7C1.txt_Green	L1 50A
US22502548_251734310065_S01_GE2_107_Sep09_2_4%7C1.txt_Red	L1 150A	US22502548_251734310065_S01_GE2_107_Sep09_2_2%7C1.txt_Green	L1 90A
US22502548_251734310065_S01_GE2_107_Sep09_2_1%7C1.txt_Red	L1 120B	US22502548_251734310065_S01_GE2_107_Sep09_2_3%7C1.txt_Red	L0 10B
US22502548_251734310065_S01_GE2_107_Sep09_2_2%7C1.txt_Red	L1 50B	US22502548_251734310065_S01_GE2_107_Sep09_1_4%7C1.txt_Green	L1 90B
US22502548_251734310064_S01_GE2_107_Sep09_2_1%7C1.txt_Green	L0 30B	US22502548_251734310065_S01_GE2_107_Sep09_1_3%7C1.txt_Green	L0 20A
