# Supplementary material for: Identification of CdnL, a Putative Transcriptional Regulator Involved in Repair and Outgrowth of Heat-Damaged Bacillus cereus Spores
Source: PLoS One. 2016 Feb 5;11(2):e0148670. doi: 10.1371/journal.pone.0148670 (PMC4746229; doi:10.1371/journal.pone.0148670)
Supplement: S2 Table — Ratios expressed are relative to untreated germinating control spores at t10. False Discovery Rates below 0.05 are indicated in bold. (PDF) [file pone.0148670.s005.pdf]

S2 Table. Expression ratios (log2 values) of DNA repair genes during germination and outgrowth of untreated and heat-treated *B. cereus* ATCC14579 spores. Ratios expressed are relative to untreated germinating control spores at t10. False Discovery Rates below 0.05 are indicated in bold

Gene	Function	Log2 values over T10 untreated						
		untreated			treated			
		T20	T30	T50	T50	T90	T120	T150
BC0026	recombination protein RecR	**1.14**	**1.23**	**0.59**	**0.57**	**0.61**	**0.59**	**0.81**
BC0057	hypothetical protein	0.41	**0.78**	**0.65**	**0.58**	**0.68**	**0.71**	**1.04**
BC0058	transcription-repair coupling factor	0.38	**1.01**	**1.34**	**1.79**	**1.79**	**1.99**	**2.19**
BC0103	DNA repair protein RadA	**0.99**	**1.79**	**3.61**	**1.19**	**2.90**	**3.43**	**3.79**
BC0468	DNA-3-methyladenine glycosylase II	**-0.63**	**-1.54**	**-2.04**	0.16	**-0.51**	**-1.08**	**-1.44**
BC0504	A/G-specific adenine glycosylase	**-0.68**	**-1.31**	**-1.62**	**-0.75**	**-1.28**	**-1.28**	**-1.43**
BC0885	3-methyladenine DNA glycosylase	**-0.58**	**-0.63**	-0.47	**0.76**	**1.03**	**0.99**	**0.92**
BC1041	ATP-dependent DNA helicase rep	**-1.20**	**-1.46**	**-2.41**	-0.09	-0.04	**-0.59**	**-1.10**
BC1137	ATP-dependent nuclease subunit B	**0.72**	**1.13**	**0.52**	**0.95**	**0.94**	**0.95**	**1.08**
BC1138	ATP-dependent nuclease subunit A	**2.19**	**3.03**	**2.26**	**1.76**	**2.17**	**2.35**	**2.71**
BC1272	DNA integration/recombination/invertion protein	**-0.74**	**-0.54**	**-0.82**	**3.12**	**3.03**	**2.60**	**1.74**
BC1363	leucine-responsive regulatory protein	**0.77**	**1.67**	**1.85**	**0.47**	**0.99**	**0.94**	**1.03**
BC1461	DNA integration/recombination/invertion protein	**0.84**	**-1.17**	**-1.83**	**2.02**	**1.47**	**0.76**	-0.11
BC1485	ATP-dependent DNA helicase recQ	0.07	-0.24	-0.46	**1.35**	**0.77**	0.03	-0.45
BC1548	endonuclease III	**-0.94**	**-1.44**	**-1.98**	**-1.09**	**-1.59**	**-1.88**	**-2.16**
BC1993	phosphohydrolase (MutT/nudix family protein)	0.09	**0.59**	0.03	**1.14**	**1.88**	**2.00**	**1.96**
BC2024	O6-methylguanine-DNA methyltransferase	**-0.91**	**-0.66**	**-1.04**	**1.40**	**1.17**	**1.04**	0.48
BC2295	exonuclease SbcD	0.07	**0.90**	**2.32**	**2.01**	**2.03**	**2.15**	**2.28**
BC2315	DinB protein	**-0.72**	**-0.67**	**1.01**	0.09	**0.48**	**0.62**	**0.83**
BC2761	phosphohydrolase (MutT/nudix family protein)	**-0.89**	**-0.99**	**-1.44**	**-0.63**	**-0.99**	**-0.87**	**-0.79**
BC2815	ATP-dependent DNA helicase recQ	**1.75**	**2.95**	**3.62**	**2.01**	**2.53**	**2.78**	**2.65**
BC3054	hypothetical protein	-0.43	0.33	**2.95**	**0.69**	**2.85**	**3.61**	**3.63**
BC3480	DNA mismatch repair protein mutS	**1.12**	**1.39**	0.25	**3.36**	**3.56**	**3.16**	**2.61**
BC3690	LexA repressor	**1.73**	**2.14**	**1.78**	**1.44**	**1.36**	**1.53**	**1.59**
BC3711	DNA integration/recombination/invertion protein	0.01	0.37	-0.42	**2.32**	**2.79**	**2.57**	**1.85**
BC3739	exodeoxyribonuclease III	**-0.55**	-0.43	**0.59**	**0.47**	**1.06**	**1.49**	**2.02**
BC3768	DNA mismatch repair protein	**1.60**	**1.65**	**0.87**	-0.23	0.39	0.11	0.30
BC3769	DNA mismatch repair protein MutS	0.22	0.15	-0.57	**-1.01**	**-0.79**	**-1.04**	**-0.95**
BC3805	polynucleotide phosphorylase	**-0.55**	-0.34	-0.22	**-1.66**	**-1.23**	-0.33	0.19
BC3832	nucleotide-binding SMF protein	**-1.16**	**-1.26**	**-1.70**	0.07	-0.42	-0.51	**-0.68**
BC3853	ATP-dependent DNA helicase RecG	-0.46	-0.07	0.37	**1.08**	**0.87**	**1.00**	**1.01**
BC3894	DnaK suppressor protein	0.03	0.29	0.38	**2.88**	**2.17**	**1.61**	**1.12**
BC4024	phosphohydrolase (MutT/nudix family protein)	-0.44	**0.78**	**2.06**	0.10	**1.03**	**1.67**	**2.29**
BC4142	DNA polymerase IV	0.31	0.06	-0.32	**1.24**	**1.28**	**1.46**	**1.05**
BC4153	phosphohydrolase (MutT/nudix family protein)	**-0.60**	**-1.42**	**-2.49**	**-0.84**	**-0.96**	**-1.35**	**-0.97**

BC4205	spore photoproduct lyase	**-0.61**	**-1.35**	**-2.08**	**3.12**	**2.50**	**1.60**	**0.90**
BC4282	endonuclease IV	**-1.60**	**-2.27**	**-3.17**	**-2.39**	**-2.79**	**-3.05**	**-3.37**
BC4295	DNA repair protein RecO	0.36	0.51	**0.87**	**2.16**	**1.47**	**1.77**	**1.91**
BC4403	single-stranded-DNA-specific exonuclease recJ	0.15	-0.15	**-1.23**	**-0.57**	**-0.85**	**-1.24**	**-1.32**
BC4447	DNA repair protein radC	**0.75**	0.28	**-0.63**	**1.64**	**1.74**	**1.30**	**0.87**
BC4461	DNA repair protein radC	**1.27**	**1.00**	0.22	**0.70**	**0.63**	**0.77**	**0.86**
BC4520	excinuclease ABC subunit C	0.22	-0.12	**-1.20**	0.31	0.13	-0.20	**-0.42**
BC4553	hypothetical protein	-0.23	**0.73**	**1.80**	**1.03**	**1.51**	**1.90**	**2.33**
BC4586	formamidopyrimidine-DNA glycosylase	0.53	**0.65**	**0.77**	**1.08**	**0.97**	**1.37**	**1.65**
BC4587	DNA polymerase I	-0.20	-0.21	0.31	**1.06**	**1.05**	**1.38**	**1.44**
BC4785	phosphohydrolase (MutT/nudix family protein)	**1.56**	**1.80**	**1.36**	**1.76**	**1.86**	**1.78**	**1.57**
BC5157	Mutator mutT protein (7,8-dihydro-8-oxoguanine-triphosphatase)	**-0.56**	**-1.02**	**-1.41**	**0.45**	**0.43**	0.04	-0.37
BC5167	excinuclease ABC subunit A	**1.62**	**1.49**	**0.91**	**0.47**	**0.56**	**1.02**	**1.31**
BC5347	UV damage endonuclease	**-1.13**	**-1.16**	**-1.83**	**1.81**	**1.25**	0.32	-0.41
BC0004	recombination protein F	not significantly different						
BC0340	DNA helicase II	not significantly different						
BC0498	recombination regulator RecX	not significantly different						
BC1020	hypothetical protein	not significantly different						
BC1223	ATP-dependent DNA helicase	not significantly different						
BC1554	Holliday junction-specific endonuclease	not significantly different						
BC1565	ATP-dependent helicase DinG	not significantly different						
BC1921	site-specific recombinase	not significantly different						
BC2032	phosphohydrolase (MutT/nudix family protein)	not significantly different						
BC2296	exonuclease SbcC	not significantly different						
BC3689	site-specific recombinase	not significantly different						
BC3740	ADA regulatory protein	not significantly different						
BC3741	O6-methylguanine-DNA methyltransferase	not significantly different						
BC3742	DNA-3-methyladenine glycosylase II	not significantly different						
BC3779	recombinase A	not significantly different						
BC4414	Holliday junction DNA helicase RuvB	not significantly different						
BC4415	Holliday junction DNA helicase RuvA	not significantly different						
BC5168	excinuclease ABC subunit B	not significantly different						
BC5398	uracil-DNA glycosylase	not significantly different						
BC5475	single-stranded DNA-binding protein	not significantly different						
BC3339	phosphohydrolase (MutT/nudix family protein)	not significantly different						
