# Supplementary material for: Identification of CdnL, a Putative Transcriptional Regulator Involved in Repair and Outgrowth of Heat-Damaged Bacillus cereus Spores
Source: PLoS One. 2016 Feb 5;11(2):e0148670. doi: 10.1371/journal.pone.0148670 (PMC4746229; doi:10.1371/journal.pone.0148670)
Supplement: S3 Table — qPCR candidate normalization genes were selected based on stable and significant expression at all time points of the microarray. Recognition sites introduced for restriction enzymes used for mutant construction are underlined in PCR primers. (PDF) [file pone.0148670.s006.pdf]

S3 Table. qPCR and PCR primers used in this study. qPCR candidate normalization genes were selected based on stable and significant expression at all time points of the microarray. Recognition sites introduced for restriction enzymes used for mutant construction are underlined in PCR primers

Primer Name / Gene ID	Sequence
**qPCR primers**	
BC0022_F	TGGTCGTTGTTAGAGGCGAT
BC0022_R	TATTAACAGGGGTTGCCGGA
BC0460_F	AATTCGTCACGCTCAATCCC
BC0460_R	TGAAGAGAGAAGAACGCAAAAACA
BC0690_F	TCGCGCGTGTTCAGTTAGAT
BC0690_R	AATCGTTTGCCTTGTAACGCC
BC0852_F	CGGAGCTGGTACGGTAGGTA
BC0852_R	AACGCCAGCTACAACCATCA
BC0853_F	CGGTGGGTTTGCTACATCCT
BC0853_R	CTGCACCTGCTGTTCCGATA
BC0854_F	TGGGAAGCTGTGTATGAGCC
BC0854_R	ATTTCACCGGCTTCTTTCGC
BC1053_F	GCAGAAAAGCTAATCGCAGATGG
BC1053_R	CGCGTAGTATTCCTTCTTGCC
BC1148_F	ACGGCAAAAGTGTAAAGACCA
BC1148_R	AGCATCATTTTGGACTGCTGTT
BC1312_F	ATTTTGACAGGGGCACGGAA
BC1312_R	GGAACGAAACTCCCAGGCAA
BC1314_F	GGCCCTCCATCGCTTGTATC
BC1314_R	TCGGCTTTTCTTCTGTTGACCA
BC1978_F	TGGTTTTGGCACAGCAGGAT
BC1978_R	GTTCCCGGTTATACGGAAGGA
BC3155_F	ACTCCACGTACCTTTTGTTTCTTT
BC3155_R	AACTTTGCCAGCGAGGACAG
BC3391_F	CGTTACCGTCATCCCCCTTC
BC3391_R	GCGATTGGTGCGTTACCTTC
BC3437_F	GCAAAGGAAGCCACGTCATC
BC3437_R	TTTTTCAGCGGGTAACGGGA
BC3438_F	AAGATCATTCAGGTGGCGGG
BC3438_R	GGTTCACTGGAAGGGTGTGT
BC3921_F1	ATGAAAAGAAACGGCGGAGAAC
BC3921_R	GCATTTGCCCGAATCCTTGT
BC3991_F	TGACACTCCTTTTGTCGCGT
BC3991_R	AGTATTCAGTTCCATATCCACCGT
BC4714_F	TCAAAACGGGGAGTCAGACC
BC4714_R	CGGCGAAGTAAATCCCGAAC
BC4834_F	GTTCAGCAATCTCACCAGCG

BC4834_R	AGCATTAGCAGATCCAACAAGGA
BC5038_F	CGTTTCTTGAGGTGGGACGA
BC5038_R	TGGAGCAGCTTGAGTGGATT
BC5242_R	ACGCCATTTACAGAGACGCC
BC5242_F	GCTCATTTTGCGCCGTTAGA
BC0544_nF	CAGAGCGTGCTGGTATTGGA
BC0544_nR	GCTTATCTGGCGGGAATGGA
BC1409_nF	TCGAAAGACGGGACGCTTG
BC1409_nR	CCACTCCTCCAGAAGCGATG
BC4743_nF	TGCTTTTGCCCACCAGATACT
BC4743_nR	TGCAAGGTGTTCCATCACGTA
BC0257_nF	CAGACGGCTCTTGCTGCTAT
BC0257_nR	AATACGCTCACCACGTACCC
BC4471_nF	CGCTGCCACTGTTCTTTCTG
BC4471_nR	AGCGATTGAGTGTCGTGAGG
**PCR primers**	
CM_fwd	CTTCCTCGAGTAAAAACCTTCTTCAACTAACGG
CM_rev	CCGGGGTACCAAAAGGATTTTTCGCTACGCTC
BC4714_up_fwd	CCTAGAATTCCAAACACCCTCTTAGCATTTCC
BC4714_up_rev	GGAGGGTACCTCGATGATTCCTGCACCGTG
BC4714_down_fwd	TTACCTCGAGATGATCAGTGAAGTTGCACTCG
BC4714_down_rev	CCGGGAATTCTTAACGTAGATGGTTGGTCAGC
BC4714_mu_up	GAAACAACCCAGCCATCTGT
BC4714_mu_down	GCTTGGTCAGGCAAATCAAT
