# Supplementary material for: Identification of CdnL, a Putative Transcriptional Regulator Involved in Repair and Outgrowth of Heat-Damaged Bacillus cereus Spores
Source: PLoS One. 2016 Feb 5;11(2):e0148670. doi: 10.1371/journal.pone.0148670 (PMC4746229; doi:10.1371/journal.pone.0148670)
Supplement: S4 Table — Counts of three independent experiments are represented individually. (PDF) [file pone.0148670.s007.pdf]

S4 Table. Number of cfu's/ml measured at day one, two and seven for untreated spores of *B. cereus* ATCC1457 and its mutant derivative strain Δ*cdnL* (BC4714) and upon exposure to wet heat, hydrogen peroxide and sodium hypochlorite treatments. Counts of three independent experiments are represented individually

Replicate	Sample	Plate count day	95°C			Hydrogen peroxide			Sodium hypochlorite		
			N [cfu/ml]			N [cfu/ml]			N [cfu/ml]		
			BHI	BHI+1.5%	BHI+5.5%	BHI	BHI+1.5%	BHI+5.5%	BHI	BHI+1.5%	BHI+5.5%
1st	wt untreated	Day 1	1.13E+08	1.50E+08		8.28E+07	1.00E+08		8.25E+07	1.11E+08	
		Day 2	1.13E+08	1.50E+08	8.33E+07	8.28E+07	1.05E+08	7.60E+07	8.25E+07	1.11E+08	1.35E+08
		Day 7	1.13E+08	1.50E+08	8.60E+07	8.28E+07	1.05E+08	7.60E+07	8.25E+07	1.11E+08	1.35E+08
	wt treated	Day 1	7.85E+06	3.83E+06		9.50E+06	1.30E+06		5.40E+06	1.45E+06	
		Day 2	7.93E+06	4.38E+06	4.75E+05	9.70E+06	1.35E+06	3.50E+04	5.53E+06	1.70E+06	1.15E+05
		Day 7	7.93E+06	4.38E+06	5.63E+05	9.70E+06	1.35E+06	3.50E+04	5.53E+06	1.70E+06	1.17E+05
2nd	wt untreated	Day 1	7.88E+07	9.55E+07		1.25E+08	1.35E+08		7.80E+07	9.83E+07	
		Day 2	7.88E+07	9.55E+07	6.15E+07	1.33E+08	1.38E+08	9.65E+07	7.80E+07	9.83E+07	1.00E+07
		Day 7	7.88E+07	9.55E+07	7.15E+07	1.33E+08	1.38E+08	9.75E+07	7.80E+07	9.83E+07	1.00E+07
	wt treated	Day 1	5.10E+06	2.60E+06		1.10E+07	2.10E+06		6.98E+06	2.03E+06	
		Day 2	5.18E+06	2.98E+06	4.38E+05	1.11E+07	2.30E+06	1.48E+05	6.98E+06	2.15E+06	1.05E+05
		Day 7	5.18E+06	2.98E+06	6.13E+05	1.11E+07	2.30E+06	1.60E+05	6.98E+06	2.15E+06	1.20E+05
3rd	wt untreated	Day 1	7.75E+07	1.53E+08		8.85E+07	1.33E+08		1.18E+08	1.15E+08	
		Day 2	7.75E+07	1.55E+08	8.90E+07	8.85E+07	1.33E+08	7.85E+07	1.20E+08	1.15E+08	7.15E+07
		Day 7	7.75E+07	1.55E+08	8.90E+07	8.85E+07	1.33E+08	8.03E+07	1.20E+08	1.15E+08	7.33E+07
	wt treated	Day 1	7.18E+06	3.73E+06		5.35E+06	9.05E+05		7.20E+06	1.03E+06	
		Day 2	7.25E+06	3.88E+06	5.95E+05	5.43E+06	9.95E+05	6.25E+04	7.20E+06	1.22E+06	7.63E+04
		Day 7	7.25E+06	3.88E+06	6.10E+05	5.43E+06	9.95E+05	6.50E+04	7.20E+06	1.22E+06	8.28E+04
1st	CdnL untreated	Day 1	7.73E+07	7.05E+07		7.93E+07	1.13E+08		7.88E+07	1.01E+08	
		Day 2	7.73E+07	7.48E+07	3.30E+07	7.93E+07	1.30E+08	7.43E+07	7.88E+07	1.01E+08	8.58E+07
		Day 7	7.73E+07	7.48E+07	3.83E+07	7.93E+07	1.30E+08	7.45E+07	7.88E+07	1.01E+08	8.70E+07
	CdnL treated	Day 1	4.20E+06	1.28E+06		8.33E+06	2.38E+06	0.00E+00	6.78E+06	1.40E+06	
		Day 2	4.20E+06	1.60E+06	1.75E+04	8.45E+06	2.48E+06	6.75E+04	6.78E+06	1.48E+06	4.75E+04
		Day 7	4.20E+06	1.60E+06	2.00E+04	8.45E+06	2.50E+06	6.75E+04	6.78E+06	1.48E+06	5.00E+04
2nd	CdnL untreated	Day 1	8.08E+07	8.43E+07		1.28E+08	1.55E+08		1.15E+08	1.23E+08	
		Day 2	8.08E+07	8.43E+07	7.53E+07	1.30E+08	1.55E+08	9.60E+07	1.15E+08	1.28E+08	7.78E+07
		Day 7	8.08E+07	8.43E+07	7.58E+07	1.30E+08	1.55E+08	9.75E+07	1.15E+08	1.28E+08	7.83E+07
	CdnL treated	Day 1	3.23E+06	3.90E+05		8.35E+06	1.65E+06		7.88E+06	2.53E+06	
		Day 2	3.60E+06	5.95E+05	3.00E+03	8.50E+06	1.65E+06	3.25E+04	7.88E+06	2.60E+06	5.50E+04
		Day 7	3.60E+06	5.95E+05	5.50E+03	8.50E+06	1.65E+06	3.25E+04	7.88E+06	2.60E+06	7.00E+04
3rd	CdnL untreated	Day 1	7.28E+07	1.78E+08		1.33E+08	1.60E+08		8.43E+07	1.13E+08	
		Day 2	7.28E+07	1.78E+08	8.15E+07	1.48E+08	1.68E+08	8.58E+07	8.43E+07	1.15E+08	8.15E+07
		Day 7	7.28E+07	1.78E+08	8.18E+07	1.48E+08	1.68E+08	8.70E+07	8.43E+07	1.15E+08	8.23E+07
	CdnL treated	Day 1	3.45E+06	8.25E+05		5.83E+06	1.10E+05		1.43E+07	2.15E+06	
		Day 2	3.65E+06	8.90E+05	1.25E+05	5.83E+06	1.55E+05	9.30E+04	1.43E+07	2.43E+06	4.38E+04
		Day 7	3.65E+06	8.93E+05	1.32E+05	5.83E+06	1.55E+05	9.38E+04	1.43E+07	2.43E+06	4.48E+04
